# Silver Nanoparticles Conjugate Attenuates Highly Active Antiretroviral Therapy-Induced Hippocampal Nissl Substance and Cognitive Deficits in Diabetic Rats

**DOI:** 10.1155/2021/2118538

**Published:** 2021-11-19

**Authors:** Sodiq Kolawole Lawal, Samuel Oluwaseun Olojede, Ayobami Dare, Oluwaseun Samuel Faborode, Edwin Coleridge S. Naidu, Carmen Olivia Rennie, Onyemaechi Okpara Azu

**Affiliations:** ^1^Discipline of Clinical Anatomy, School of Laboratory Medicine & Medical Sciences, Nelson R Mandela School of Medicine, University of KwaZulu-Natal, 719 Umbilo Road, Durban, South Africa; ^2^Department of Physiology, School of Laboratory Medicine & Medical Sciences, Westville Campus, University of KwaZulu-Natal, Durban, South Africa; ^3^Department of Anatomy, School of Medicine, University of Namibia, Windhoek, Private Bag 13301, Namibia

## Abstract

**Background:**

The application of nanomedicine to antiretroviral drug delivery holds promise in reducing the comorbidities related to long-term systemic exposure to highly active antiretroviral therapy (HAART). However, the safety of drugs loaded with silver nanoparticles has been debatable. This study is aimed at evaluating the effects of HAART-loaded silver nanoparticles (HAART-AgNPs) on the behavioural assessment, biochemical indices, morphological, and morphometric of the hippocampus in diabetic Sprague-Dawley rats.

**Methods:**

Conjugated HAART-AgNPs were characterized using FTIR spectroscopy, UV spectrophotometer, HR-TEM, SEM, and EDX for absorbance peaks, size and morphology, and elemental components. Forty-eight male SD rats (250 ± 13 g) were divided into nondiabetic and diabetic groups. Each group was subdivided into (*n* = 8) A (nondiabetic+vehicle), B (nondiabetic+HAART), C (nondiabetic+HAART-AgNPs), D (diabetic+vehicle), E (diabetic+HAART), and F (diabetic+HAART-AgNPs). Morris water maze, Y-maze test, and weekly blood glucose levels were carried out. Following the last dose of 8-week treatment, the rats were anaesthetized and euthanized. Brain tissues were carefully removed and postfixed for Nissl staining histology.

**Results:**

1.5 M concentration of HAART-AgNPs showed nanoparticle size 20.3 nm with spherical shape. HAART-AgNPs revealed 16.89% of silver and other elemental components of HAART. The diabetic control rats showed a significant increase in blood glucose, reduced spatial learning, positive hippocampal Nissl-stained cells, and a significant decrease in GSH and SOD levels. However, administration of HAART-AgNPs to diabetic rats significantly reduced blood glucose level, improved spatial learning, biochemical indices, and enhanced memory compared to diabetic control. Interestingly, diabetic HAART-AgNP-treated rats showed a significantly improved memory, increased GSH, SOD, and number of positive Nissl-stained neurons compared to diabetic-treated HAART only.

**Conclusion:**

Administration of HAART to diabetic rats aggravates the complications of diabetes and promotes neurotoxic effects on the experimental rats, while HAART-loaded silver nanoparticle (HAART-AgNP) alleviates diabetes-induced neurotoxicity.

## 1. Introduction

Diabetes mellitus is a chronic metabolic disorder characterized by hyperglycaemia. The long-term effects of diabetes can lead to conditions such as neuropathy, nephropathy, and cardiovascular complications [1, 2]. Type 2 diabetes mellitus (T2DM) accounts for 90-95% of diabetic conditions in people living with diabetes. Type 2 diabetic patients have insulin resistance and usually insulin deficiency but may not require insulin injection throughout their lifetime to survive [1].

Many drugs cause insulin resistance or impaired insulin secretion, leading to diabetes in individuals with risk factors. The association between highly active antiretroviral therapy (HAART) and the development of T2DM has been reported [3, 4].

Diabetes mellitus has been implicated in various neurological diseases such as neuropathy and neuroinflammation. Its long-term effects on the brain can manifest at physiological and structural levels [5]. In diabetic subjects and experimental rats, cognitive dysfunction has been reported in various literature [6–8].

The standard HAART comes in a combination form of two nucleoside reverse transcriptase inhibitors (emtricitabine or lamivudine with one of tenofovir, zidovudine, or abacavir) and with one of the nonnucleoside reverse transcriptase inhibitors (efavirenz) or protease inhibitors (ritonavir/atazanavir) or integrase inhibitor (raltegravir) [9].

The HAART, a combination of tenofovir (TDF), emtricitabine (FTC), and efavirenz (EFV) as a fixed-dose therapy for the treatment of Human Immunodeficiency Virus (HIV), has proven to be more effective when compared to other fixed-dose combinations [10]. While HAART has improved the quality of life of HIV patients [11], the long-term treatment predisposes patients on HAART to the development of other secondary derangements like neurological diseases, endocrine disorders (diabetes mellitus), and heart diseases [12, 13]. The long-term effect of HAART is associated with chronic metabolic disorders like diabetes mellitus and dyslipidemia [14]. The metabolic disorders emerging from the combined antiretrovirals (cARVs) are associated with long-term exposure of the entire body to multiple drugs at high doses leading to drug toxicity [15].

The mechanism through which HAART causes diabetes is not fully understood. However, a study by Avari and Devendra [16] has attributed the cause to cell toxicity and oxidative stress due to long systemic exposure. HIV-1 infection alone or in combination with HAART has been implicated in increase chemically reactive species in circulation by producing oxidized metabolites through the interaction between ROS and infected biomolecules [17–19]. Similarly, highly active antiretroviral therapy has been implicated in mitochondrial damage and subsequently increases the risk of neuropathy, retinopathy, and nephropathy [20, 21]. The hippocampus plays a significant role in learning and memory, and it has been linked with neuronal vulnerability due to oxidative stress [22]. In addition, pyramidal neurons in the hippocampal CA1 are more vulnerable to massive cell death compared to other hippocampal regions when exposed to oxidative stress generating agents [23].

The application of nanobased drug delivery systems in the treatment of diseases including HIV/AIDS is relatively new but rapidly developing, where nanoparticles are employed as a vehicle to deliver the therapeutic agent to specifically targeted cells in a controlled manner [24].

The emergence of the drug nanodelivery system has tremendous improvement on HIV/AIDS treatment because of its ability to achieve drug efficacy, crossing biological barriers, targeting specific cell populations, and long-term drug release [25, 26]. Nevertheless, the issue related to toxicity has been reported in some of the antiretroviral drugs coupled with nanoparticles. Consequently, this has raised a concern about the safety of the nanoparticle drug delivery system [15, 27].

Silver nanoparticles (AgNPs) have been used for a wide range of applications in the biomedical field due to their novel property and simple synthesis method [28]. AgNPs have been utilized for delivering therapeutic agents such as antiviral, antibiotics, antifungal, and chemotherapeutic agents [29].

Hence, this study is aimed at evaluating the effects of HAART-loaded silver nanoparticles (HAART-AgNPs) on the behavioural assessment, biochemical indices, and morphological and morphometric of the hippocampus in STZ-induced diabetic Sprague-Dawley rats.

## 2. Materials and Methods

### 2.1. Chemical and Highly Active Antiretroviral Therapy

The highly active antiretroviral therapy (HAART), a fixed-dose combination of efavirenz (EFV, 600 mg), emtricitabine (FTC, 200 mg), and tenofovir disoproxil fumarate (TDF, 300 mg), was bought from Dis-Chem Pharmacy Ballito, South Africa. The other chemicals such as silver nitrate (AgN0_3_), trisodium citrate, streptozotocin (STZ), and sodium hydroxide of analytical grade were bought from Sigma-Aldrich Company, Johannesburg, South Africa. All the chemicals were of analytical quality. Sterile distilled water was used in all the stages of this experiment throughout.

### 2.2. Synthesis of AgNPs

Silver nanoparticles were synthesized according to Turkevich et al. [30]. Silver nitrate (AgNO_3_) was used as a template, trisodium citrate (TSC) was used as a reducing and stabilizing agent, and distilled deionized water was used as a dissolving agent. An aqueous solution of 0.30 M AgNO_3_ was prepared by drying the AgNO_3_ crystal in an oven at 100°C, weighing 5.10 g of the dried crystals in a 100 mL volumetric flask, and dissolving double-distilled water. A series of TSC solutions (0.5 M, 1 M, 1.5 M, and 2 M) was prepared from an aqueous stock solution of 2 M (147 g in 250 mL of double-distilled water). Four TSC solutions of varying concentrations (0.5 M, 1 M, 1.5 M, and 2 M) were used for the study. The AgNPs formed were under continuous stirring at 90°C for 5 minutes at a pH of 10.5. After that, the solution changed in colour from colourless to amber, signifying the formation of AgNPs.

### 2.3. Formulation of HAART-AgNPs

15 g of the HAART was weighed out and dissolved in 10 mL concentrated sodium hydroxide, put in a 50 mL volumetric flask, and added distilled water. The 50 mL HAART solution was mixed with 100 mL AgNP aqueous solution. The concentration of HAART used was 1.05 M. The ultrasonicator was used to ensure the proper blending of the constituents, the AgNPs, and HAART.

The resultant nanoformulations, HAART-AgNPs, were centrifuged at 4,500 rpm at 40°C, at 40 minutes to separate the unincorporated drug. The supernatant was analyzed using a UV spectrophotometer at a wavelength of 285-315 nm to calculate the quantity of unincorporated drug (W1) from the total amount of drug coupled with silver nanoparticle (W2).

The percentage incorporated efficiency for the formulated HAART-AgNPs was estimated using

(1) $$\begin{array}{c} \%\text{IE}=\frac{\text{W}2-\text{W}1}{\text{W}1}\times 100=90.52\pm 0.5\%. \end{array}$$

### 2.4. Characterization of AgNPs and HAART-AgNPs

The formulated nanoconjugates (AgNPs and HAART-AgNPs) were confirmed by ultraviolet-visible (UV-Vis) spectroscopy (Shimadzu MultSpec-1501, Shimadzu Corporation, Tokyo, Japan) and Fourier transform infrared (FTIR) spectroscopy (Perkin-Elmer Universal ATR spectrometer, USA). The morphology and size of the nanoparticles were evaluated by a high-resolution transmission electron microscope (HR-TEM, JEOL 2100, Japan) operated at a voltage of 200 Kv. Field emission scanning electron microscope (FESEM, Carl Zeiss, Germany) performed at a voltage of 5 keV with energy dispersive X-ray (EDX, Aztec Analysis Software, England).

### 2.5. Experimental Animal

Forty-eight [31] adult male Sprague-Dawley rats weighing (250 ± 13 g) were obtained from the Biomedical Research Unit (BRU) of the University of KwaZulu-Natal. All the animals were housed in plastic cages (4 rats/cage) of 30 cm length, 25 cm width, and 15 cm height in the animal room of the BRU. The animals were allowed free access to water and feed (standard rat pellets), and the animal laboratory room was maintained at a temperature of 26-28°C and 12 : 12 light : dark cycle.

The animals were handled in accordance with the standard guides for the animal laboratory, and the University of KwaZulu-Natal Animal Ethics Committee approved the protocol (AREC/044/019D).

### 2.6. Induction of Experimental Type-2 Diabetes Mellitus

Type 2 diabetes mellitus was induced in the experimental animals according to Wilson and Islam [32]. Briefly, each rat received 10 g of fructose dissolved in 100 mL of water (10% fructose solution *ad libitum*) for two weeks followed by an overnight fasting and single intraperitoneal injection of STZ (40 mg/kg B.W) in 0.9% NaCl with 100 mM sodium citrate buffer (pH 4.5). The control rats received the vehicle (citrate buffer).

The diabetic condition was confirmed five days after STZ injection by blood glucose level ≥ 200 mg/dL.

### 2.7. Drug Administration and Grouping

Drug administration began after the rats were confirmed diabetic. The rats were either administered vehicle, HAART, or HAART-AgNPs once daily using oral cannula except for HAART-AgNPs, which was administered intraperitoneally (5 days/week).

The animals were grouped and treated for eight [8] weeks as follows:

Group A: nondiabetic control+vehicle (0.5 mL/100 g)

Group B: nondiabetic+HAART (98.2 mg/kg b.w oral)

Group C: nondiabetic+HAART-AgNPs (24.5 mg/kg b.w i. p)

Group D: diabetic control+vehicle (0.5 mL/100 g)

Group E: diabetic+HAART (98.2 mg/kg b.w oral)

Group F: diabetic+HAART-AgNPs (24.5 mg/kg b.w i. p)

Everson et al. [33].

### 2.8. Behavioural Assessment

#### 2.8.1. Morris Water Maze Task for Testing the Spatial Learning and Memory

The rats' spatial learning and memory performance were evaluated using a Moris water maze, as previously described by Greish et al. and Morris [34, 35]. The Moris water maze apparatus consists of a standard circular swimming pool of 100 cm in diameter. The pool is half-filled with water maintained at a temperature of 22-25°C. The rats were trained four times a day from the predetermined different quadrant of the pool with an interval of 20 mins for three consecutive days. Each rat was given a maximum of 120 seconds to find the hidden platform submerged 2 cm below the water level, and those animals that failed to locate the escape platform during the 120 seconds of the trial time were gently guided to the platform and left there for 20 seconds.

The latency to reach the hidden platform submerged 2 cm below the water level was recorded.

Two days later, the probe trial was performed on each animal, following the same procedure as the training trial. In the probe trial, the hidden platform was removed from the pool, but other cues remained in the same place for both training trials and probe trials, and animals were given 120 seconds of swimming in searching for the platform. The starting point for both the training trial and probe trial is the marked point in the quadrant opposite the platform quadrant, and this was used for the probe trial recording. The percentage of time spent swimming in the quadrant previously contained platform was recorded and calculated. The longer the time spent in the quadrant that previously had the platform was considered as a measure of learning and memory retention.

#### 2.8.2. Y-Maze Task for Testing the Working Memory, Learning, and Locomotion

The spontaneous alternation was used to measure the spatial working memory and locomotion activity according to Hughes and Typlt et al. [36, 37]. Briefly, Y-maze is composed of three equal arms measured 120° interconnected with one another. The arms were 40 cm long, 15 cm high, and 10 cm wide.

Each arm is identified by letters A, B, and C. The rats were placed at the centre of the maze and allowed to move freely for 5 mins, and their tails must completely enter into the arm from the centre before recording as one entry.

The number of entries was recorded using a video camera, and two independent observers recorded the data from the computer system. Between each experiment session, the apparatus was cleaned with a tissue soaked with alcohol and, after that, dry with tissue paper.

Percent alternation was calculated as follows:

(2) $$\begin{array}{c} \%\text{Alternation}=\left(\frac{\text{Number of Alternations}}{\left[\text{Total number of arm entries}–2\right]}\right)\times \left.100\right), \end{array}$$

by Typlt et al. [37]

### 2.9. Biochemical Analysis

#### 2.9.1. Tissue Preparation

The brain tissue was harvested and rinsed in PBL. 0.5 g of the harvested brain tissue was homogenized in 5 mL sodium phosphate buffer with 1% triton with 1% triton X-100 (50 mM; pH 7.5). The homogenates were centrifuged for 10 mins at 20,000 g (4°C). The supernatants were decanted into 2 mL Eppendorf tubes, labelled, and preserved at -80°C until further analysis.

#### 2.9.2. Determination of Oxidative Stress Markers

The brain homogenates were used to measure the concentration of reduced glutathione (GSH), antioxidant enzymes (SOD), and lipid peroxidation (MDA) using a spectrophotometric assay.

Glutathione (GSH) level was assessed according to Ellman's [38] protocol, Superoxide dismutase (SOD) activity was determined according to Kakkar et al. [39], and malondialdehyde (MDA) was determined using the protocol of Mkhwanazi et al. [40].

#### 2.9.3. Histological (Nissl Staining) for Brain Tissue

All the rats from each group were anaesthetized and euthanized at the end of the experiment, followed by the total body perfusion with 0.1 M phosphate buffer saline (pH 7.4) transcardially. The brain was carefully removed and weighed, then postfixed in 4% paraformaldehyde for 48 h. The hippocampus of the rat was dissected from the brain and processed for paraffin embedding. Following this, the brain tissues were infiltrated with 30% sucrose for three days at 4°C. The paraffin-embedded brain tissues were sectioned at 5 𝜇m using Leica RM 2255 microtome, and these tissues were stained according to the method of Gu et al. [41]. The coronal sections (5 𝜇m) of the hippocampus were cut and submerged in 0.1% cresyl violet for 10 min at 37°C and then rinsed in distilled water. It was then dehydrated in graded ethanol (50%, 70%, 90%, and 100%) and cover lipped with neutral balsam for morphological and morphometric studies. The number of positive cells in the hippocampal CA1 area was used for morphometric analysis. The counting of cells was done by the investigator blinded to the protocol. All procedures were triplicated, and data were represented as cell count per mm^2^.

### 2.10. Statistical Analysis

Most measurements are continuous variables and are presented graphically by descriptive statistics. ANOVA was used to compare more than two groups to determine the statistical significance. Where one-way ANOVA was used, Dunnett's multiple comparison post hoc test was carried out. All analyses were done using GraphPad Prism 9 for Windows (GraphPad Software 2365 Northside Dr Suite 560 San Diego, CA 92108), and values were expressed as mean ± SEM. *p* < 0.05 was considered statistically significant.

## 3. Results

### 3.1. The Characterization of AgNPs and HAART-AgNPs

#### 3.1.1. Fourier-Transform Infrared (FTIR) Spectroscopy

**Figure 1:**
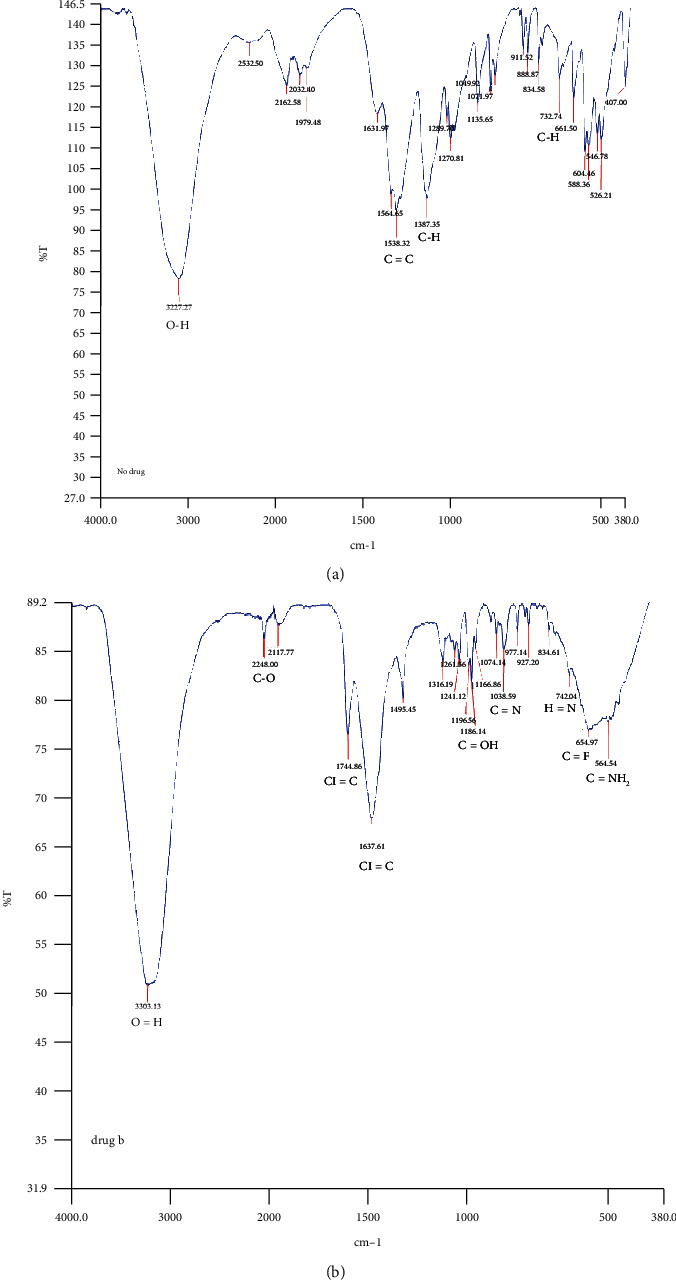
IR spectra of (a) AgNPs and (b) HAART (efavirenz, emtricitabine, and tenofovir disoproxil fumarate).

As presented in Figure 1, FTIR spectroscopy was used to identify the various functional groups in the nanoconjugates responsible for reducing Ag^+^ to Ag^0^ and stabilizing the AgNPs. The IR spectrum of AgNPs shows absorption bands at 3227.27 cm^−1^ (Figure 1(a)) while HAART shows absorption bands at 3303.13 cm^−1^ (Figure 1(b)); both of these bands are ascribed to O-H stretching vibration. The IR spectrum of AgNPs shows an absorption band at 1387.35 cm^−1^ on C-H stretching vibration due to the presence of TSC on the surface of the nanoparticles. The IR spectrum of HAART reflects absorbance at various stretching vibrations based on the presence of functional groups (O-H 3303.13 cm^−1^, C=O 2248.60, C=C 1744.86, Cl=C 1637.61, C-OH 1185.14, C=N 1038.59, H-N 742.04, C=F 654.97, and C=NH_2_ 564.54).

**Figure 2:**
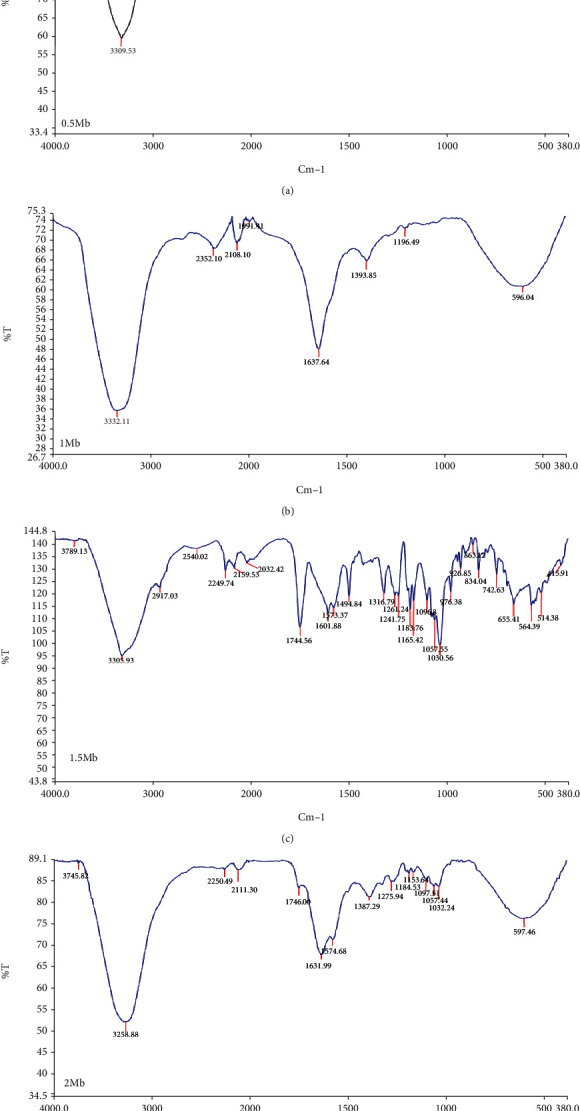
IR spectra of (a) HAART-AgNPs (0.5 M), (b) HAART-AgNPs (1 M), (c) HAART-AgNPs (1.5 M), and (d) HAART-AgNPs (2 M).

The IR spectra for HAART-AgNPs show the stretching vibrations of O-H functional group main characteristics as indicated in Figure 2 based on the concentrations: 0.5 M = 3309.53 cm^−1^ (Figure 2(a)), 1 M = 3332.11 cm^−1^ (Figure 2(b)), 1.5 M = 3305.93 cm^−1^ (Figure 2(c)), and 2 M = 3258.88 cm^−1^ (Figure 2(d)).

### 3.2. High-Resolution Transmission Electron Microscopy (HR-TEM)

**Figure 3:**
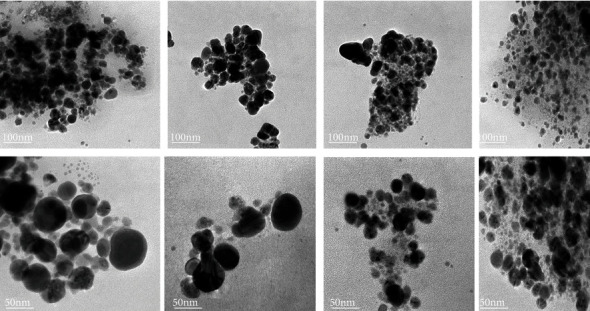
HR-TEM images of HAART-AgNPs (0.5 M) (A1 and A2), HAART-AgNPs (1 M) (B1 and B2), HAART-AgNPs (1.5 M) (C1 and C2), and HAART-AgNPs (2 M) (D1 and D2).

**Figure 4:**
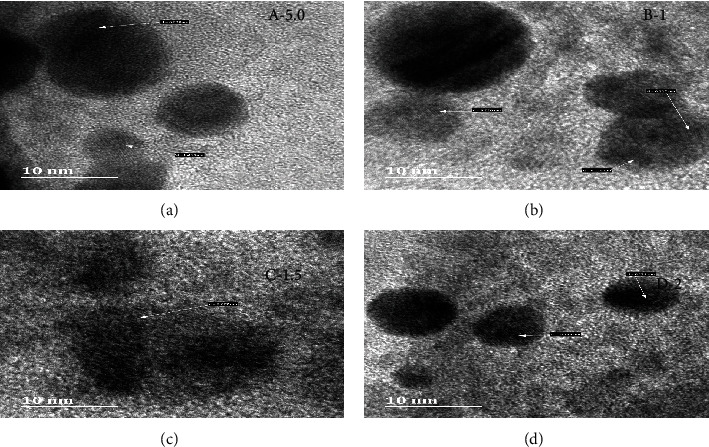
HR-TEM images of (a) HAART-AgNPs (0.5 M), (b) HAART-AgNPs (1 M), (c) HAART-AgNPs (1.5 M), and (d) HAART-AgNPs (2 M) with interplanar spacing (d).

As shown in Figures 3 and 4, the size and morphology of the newly formulated nanoconjugate (HAART-AgNPs) were investigated by HR-TEM. The investigation revealed the particle size for each of the concentrations as follows: HAART-AgNPs (0.5 M) = 20.3 nm, 1 M = 19.2 nm, and 1.5 M = 19.4 nm, and the 2 M gave the particle size of 32.3 nm. The shape of the formulated HAART-AgNPs was spherical for all the concentrations. Also, the HAART-AgNPs also revealed monodispersed and polydisperse particles.

HR-TEM images also revealed the lattice fringes of the conjugated HAART-AgNPs of different concentrations. The lattice fringe of 0.238 nm is for 0.5 M HAART-AgNPs, 0.224 for 1 M HAART-AgNPs, 0.277 nm for 1.5 nm, and 0.234 nm for 2 M. The HAART-AgNPs displayed a d-spacing corresponding to the plane of the cubic structure.

### 3.3. SEM for Morphology and Structure

**Figure 5:**
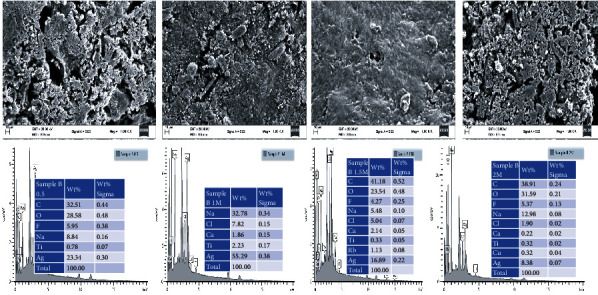
SEM images and EDX spectra of HAART-AgNPs (0.5 M) (A1 and A2), HAART-AgNPs (1 M) (B1 and B2), HAART-AgNPs (1.5 M) (C1 and C2), and HAART-AgNPs (2 M) (D1 and D2), respectively.

The morphology and structure of the conjugated HAART-AgNPs were investigated by SEM (Figure 5). The SEM demonstrated spherical shape particles for 0.5 M, 1 M, and 1.5 M and the mixture of spherical and a few hexagonal for 2 M as it was shown in the TEM (Figure 5). The SEM displayed a nanorod covered with nanosphere structure for 0.5 M, 1 M.

### 3.4. EDX for Confirmation of Functional Group

The elemental constituents of the formulated HAART-AgNPs were confirmed by EDX. The results show a peak of approximately 3 keV for all the concentrations. A similar finding documented a peak of around 3 keV for the AgNPs synthesized. Also, the result in this study indicated that 1 M exhibited the highest percentage of silver nitrate (Ag) with a total weight of 55.2 g percentage of silver nitrate. In addition, a concentration of 1.5 M contained 16.89% of Ag, and 23.34% of Ag was discovered in 0.5 M, while 2 M concentration possessed the least Ag percentage with 8.38%.

### 3.5. HAART-AgNPs Reduced Blood Glucose Level in Diabetic Condition

**Figure 6:**
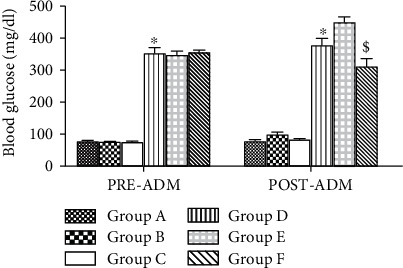
Blood glucose level for pre- and postadministration in nondiabetic groups: vehicle (A), HAART (B), and HAART-AgNPs (C), and diabetic groups administered with vehicle (D), HAART (E), and HAART-AgNPs. ^∗^*p* < 0.05 vs. group A (nondiabetic control); ^$^*p* < 0.05 vs. group E (diabetic+HAART).

There was a significant (*p* < 0.05) increase in blood glucose level in diabetic groups compared to their respective control in pre- and postadministration. Interestingly, after 8 weeks of treatment, group F (diabetic+HAART-AgNPs) shows a significant (*p* < 0.05) reduction in blood glucose level compared to group E (diabetic+HAART), as shown in Figure 6.

### 3.6. HAART-Loaded Silver Nanoparticles (HAART-AgNPs) Enhance Learning in Diabetic Rats

**Figure 7:**
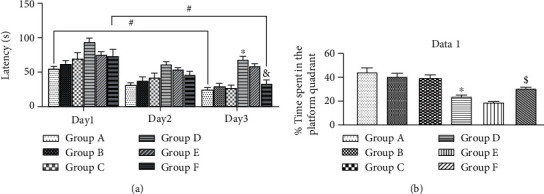
(a) Moris water maze (latency for 3-day training trails) for nondiabetic and diabetic treated rats. Nondiabetic groups administered with water (A), HAART (B), and HAART-AgNPs (C) and diabetic groups administered with water (D), HAART (E), and HAART-AgNPs, a = Figure 1(a). Data are expressed as mean ± SEM (*n* = 8); ^∗^*p* < 0.05 vs. group A (nondiabetic control), ^#^*p* < 0.05 vs. day 1, ^&^*p* < 0.05 vs. group D (diabetic control). (b) Morris Water Maze (% time spent in the platform quadrant) for nondiabetic groups administered with vehicle (A), HAART (B), and HAART-AgNPs (C) and diabetic groups administered with vehicle (D), HAART (E), and HAART-AgNPs, b = Figure 1(b). Data are expressed as mean ± SEM (*n* = 8); ^∗^*p* < 0.05 vs. group A (nondiabetic control); ^$^*p* < 0.05 vs. group E (diabetic+HAART).

Spatial learning was assessed in nondiabetic and diabetic groups treated with vehicle, HAART, and HAART-AgNPs using Morris water maze (latency to reach the hidden platform 2 cm below water level). The control group A shows learning behaviour by significantly reducing the latency to reach the submerged platform in the pool in four trials for three consecutive days *p* < 0.05. Group F (diabetic+HAART-AgNPs) shows a significant reduction in the latency to reach the hidden platform between the first and third-day trials. Interestingly, diabetic rats treated with HAART-AgNPs significantly enhanced learning by reducing the latency compared to diabetic control group D on the last day trails, as shown in Figure 7.

### 3.7. HAART-AgNPs Improved Memory in Diabetic Condition

The percentage of time spent swimming in the quadrant previously contained platform was significantly reduced in group D (diabetic control) compared to group A (nondiabetic control). Administration of HAART-AgNPs to the diabetic rats significantly (*p* < 0.05) improved memory by increasing the time spent in the quadrant previously contained platform compared to diabetic control (group D), as shown in Figure 7(b).

### 3.8. HAART-AgNPs Significantly Improved Spatial Working Memory in Diabetic Rats

**Figure 8:**
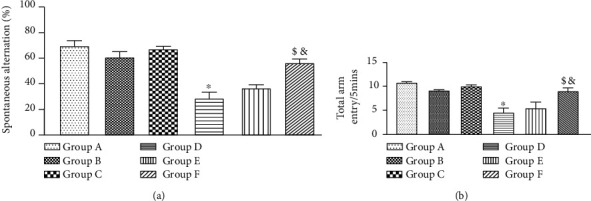
(a) Y-maze (spontaneous alternation) for nondiabetic groups administered with vehicle (A), HAART (B), and HAART-AgNPs (C) and diabetic groups administered with vehicle (D), HAART (E), and HAART-AgNPs, a = (a). ^∗^*p* < 0.05 vs. group A (nondiabetic control); ^&^*p* < 0.05 vs. group D (diabetic control); ^$^*p* < 0.05 vs. group E (diabetic+HAART). (b) Y-maze (locomotion) for nondiabetic groups and diabetic groups. Nondiabetic groups administered with vehicle (A), HAART (B), and HAART-AgNPs (C) and diabetic groups administered with vehicle (D), HAART (E), and HAART-AgNPs, b = Figure 8(b). Data are expressed as mean ± SEM (*n* = 8); ^∗^*p* < 0.05 vs. group A (nondiabetic control), ^&^*p* < 0.05 vs. group D (diabetic control), ^$^*p* < 0.05 vs. group E (diabetic+HAART).

Working memory was assessed on nondiabetic and diabetic groups treated with vehicle, HAART, and HAART-AgNPs using Y-maze. Diabetic control (group D) shows a significant (*p* < 0.05) reduction in spontaneous alternation compared to nondiabetic control (group A). There was a significant improvement in the spatial working memory of diabetic rats treated with HAART-AgNPs (group F) compared to diabetic groups treated with HAART (group E) and diabetic control (group D), as shown in Figure 8(a).

### 3.9. Diabetic and Highly Active Antiretroviral Therapy Reduced Locomotion While HAART-AgNPs Improved Locomotion

Locomotion was evaluated using a total number of arm entries/5 mins in the Y-maze. Locomotion activity was significantly reduced (*p* < 0.05) in diabetic control compared to nondiabetic control group A. Interestingly, group F (diabetic+HAART-AgNPs) improved locomotion compared to group D (diabetic control) and group E (diabetic+HAART), as shown in Figure 8(b).

### 3.10. Oxidative Stress Markers

There was a significant decrease (*p* < 0.05) in the SOD and GSH levels of diabetic control (group D) compared to nondiabetic control (group A). MDA also significantly increased in the diabetic control compared to nondiabetic control. Notably, there was a significant (*p* < 0.05) improvement in GSH, and MDA levels of the diabetic rats treated with HAART-AgNPs (group F) compared to diabetic rats treated with HAART only (group E), as presented in Table 1.

### 3.11. HAART-AgNPs Increased the Positive Cell Nissl Staining

**Figure 9:**
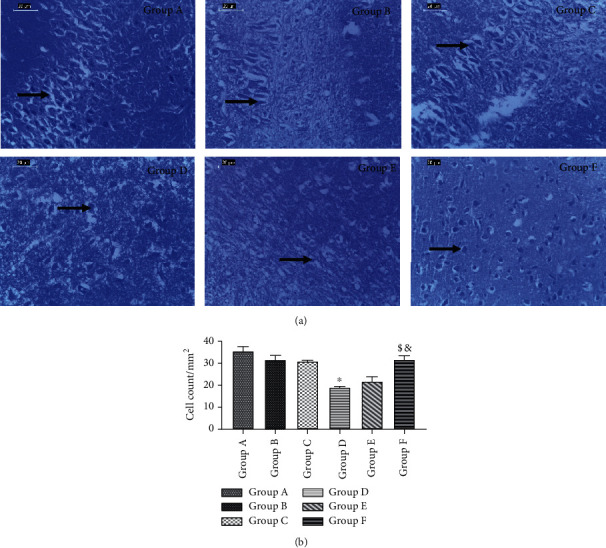
(a) Hippocampus CA1 Nissl staining histology (×400). A = nondiabetic control showing distinct pyramidal cells with well-stained Nissl bodies. B = nondiabetic+HAART showing pyramidal cells with mildly stained Nissl bodies. C = nondiabetic+HAART-AgNPs showing pyramidal cells with distinct stained Nissl bodies. D = diabetic control showing necrotic pyramidal cells with poorly stained Nissl bodies. E = diabetic+HAART showing pyramidal cells with mildly stained Nissl bodies and vacuolated neurons. F = diabetic+HAART-AgNPs showing pyramidal cells with distinct stained Nissl bodies. (b) Population of Nissl-stained neurons in the hippocampal CA1. Data are expressed as mean ± SEM (*n* = 8); ^∗^*p* < 0.05 vs. group A (nondiabetic control), ^&^*p* < 0.05 vs. group D (diabetic control); ^$^*p* < 0.05 vs. group E (diabetic+HAART).

The Nissl staining of hippocampal CA1 shows the various degree of necrotic pyramidal cells in all diabetic groups. The diabetic group administered with HAART (group D) shows poorly Nissl staining compared with the nondiabetic control group. Interestingly, the Nissl staining for the diabetic group treated with HAART-AgNPs (group F) shows improved Nissl staining of the pyramidal cells, as shown in Figure 9.

### 3.12. HAART-AgNPs Increased the Number of Surviving Positive Stained Neurons

There was a significant (*p* < 0.05) decrease in positive Nissl-stained neurons in diabetic control (18.62 ± 0.866) compared to nondiabetic control (35.08 ± 2.377). Notably, diabetic rats treated with HAART-AgNPs (31.30 ± 2.024) showed a significant increase *p* < 0.05 in the number of surviving positively stained neurons compared to diabetic control and diabetic-treated HAART (21.40 ± 2.494) as shown in Figure 9(b).

## 4. Discussion

**Figure 10:**
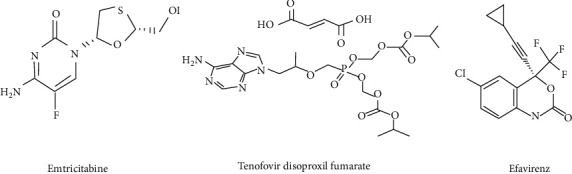
Structure of emtricitabine, efavirenz, and tenofovir disoproxil fumarate showing various functional groups present in each drug component of HAART. Adapted from [42].

This study shows the synthesis of various sizes of AgNPs using silver nitrate as a template and trisodium citrate as stabilizing and reducing agent. Highly active antiretroviral therapy (HAART) (Figure 10) was loaded on the surface of the AgNPs. After that, the functional groups, absorbance peaks, size and morphology, and elemental components of formulated (HAART-AgNPs) were studied using FTIR spectroscopy, UV spectrophotometer, HR-TEM, SEM, and EDX.

In the present study, the formulated HAART-AgNPs showed a percentage incorporation efficiency of 90.52 ± 0.5%. This indicated that a more significant amount of the HAART was incorporated with silver nanoparticles.

The IR spectra for the incorporated HAART-AgNPs revealed different absorbances at the O-H functional group after the incorporation. These changes in the stretching vibrations of the O-H group at various concentrations indicate stabilizing agents on the nanoparticles. A study by [43] observed the stretching vibration of the O-H group at 3300-3500 cm^−1^ which was within the range of our observations at different concentrations of the stabilizing agent. In addition, this result indicated that the structural components of HAART were not disorganized despite silver nanoparticle interaction.

Our formulated HAART-AgNPs revealed a spherical shape and mean nanoparticle sizes between 19 and 32 nm in all concentrations. Spherical shapes with nanoparticle size between 20 and 50 nm of nanoparticles have been described as not toxic to the biological tissue [44–46]. The previous literature has reported that size and shape are the primary determinants of toxicity in silver nanoparticles [47].

The SEM displayed a nanorod covered with nanosphere structure for 0.5 M, 1 M, and 1.5 M. This finding agrees with a study by [44] that reported a fabricated AgNPs of nanorod surrounded by nanosphere with different concentrations of AgNPs for drug delivery.

Finally, the elemental constituents of the formulated HAART-AgNPs were confirmed by EDX, the result in this study indicated that the concentration of 1.5 M formulated HAART-AgNPs has moderate silver (16.89%), and the presence of elemental compositions such as oxygen (O), carbon (C), fluorine (F), and chlorine (Cl) indicates that HAART was fully incorporated with AgNPs. Studies have shown that the elemental compositions of the drugs must be shown in the conjugated nanodrug to establish the proper incorporation [46, 48]. The remaining elemental composition like sodium, which was derived from TSC, titanium, rubidium, and calcium, was derived from copper grid used for sample preservation.

Based on the toxicity criteria as reported in the literature ([44–46]) such as particle size and shape, elemental compositions of this presence conjugated HAART-AgNPs with 1.5 concentration were used for animal studies.

Diabetes mellitus in people living with HIV is associated with various neurological complications [6, 8, 31]. The incidence of diabetes mellitus is up to four times more common in HIV-infected men exposed to HAART than in men living with HIV without treatment [49]. A recent study showed that antiretroviral drugs coupled with nanoparticles hold a promising future in reducing the adverse effect of HAART while achieving maximum drug efficacy [27]. However, the safety of antiretroviral therapy-loaded nanoparticles is debatable [50, 51].

In the present study, daily oral administration of HAART to diabetic animals further increases the blood glucose levels in diabetic rats. This observation shows that HAART has metabolic effects by increasing the blood glucose level in experimental rats, supporting the findings of Maganga et al. [52], who reported the five folds of glucose metabolism disorder in HIV-infected adults on a long-term HAART. However, the diabetic rats treated with HAART-loaded silver nanoparticles showed a decrease in blood glucose level after eight weeks of treatment which may be due to the reduced quantity of HAART in the silver-loaded nanoparticle at eight weeks and glycaemic effects of silver nanoparticles. This finding corroborates the report of Alkaladi et al. [53] who observed that silver nanoparticle acts as a potent antidiabetic agent due to its glycaemic effects.

In this study, rats administered with HAART failed to show differences between day one and day three latency trials to reach the submerged platform. This result suggests impairment in spatial learning and memory in the diabetic group treated with HAART. The evidence regarding the potential neurotoxicity of HAART related to cognitive impairment has been reported [54]. HAART with higher central nervous system penetration is associated with increased neurotoxicity, thus improving cognitive impairment [55].

Interestingly, HAART-coupled silver nanoparticle conjugate (HAART-AgNPs) showed a consistent spatial learning pattern by significantly reducing the latency to reach the hidden platform in the three-day trials.

The HAART-AgNPs have high CNS penetration-effectiveness due to their side (10-100 nm), enhanced bioavailability, and reduced CSF viral loads while reducing toxicity profile [56].

An investigation on the spatial working memory of the diabetic rat treated with HAART showed significant learning and memory deficit. The memory deficits observed in this study may result from the diabetogenic effects of HAART on the experimental rats by increasing the blood glucose level in diabetic rats. Despite the benefit of HAART, changes in the brain cytoarchitecture and related impairments on spatial memory and learning have been observed in experimental animals and normal HIV-positive individuals [57, 58]. However, HAART-silver nanoparticle conjugate (HAART-AgNPs) showed an improvement in spatial working memory in this study. The memory improvement observed in the HAART-AgNP diabetic treated group may be due to a reduction in HAART dosage when coupled with silver nanoparticles. Also, silver nanoparticles have been reported to act as an antidiabetic agent [53].

HAART-AgNPs significantly improved locomotion activity in diabetic rats compared to HAART-treated only. This reduction in locomotion seen in diabetic rats treated HAART may be related to anxiety-like behaviour. This finding corroborates the report of Gyang et al. [59], who observed locomotion deficits in *Drosophila melanogaster* treated with EFVb-HAART and possible neurotoxic consequences.

Oxidative stress (OS) forms the biological basis for many diseases, and it plays a vital role in neuronal injury [60]. In this study, an increase in MDA level and a reduction in the production of SOD and GSH were observed in diabetic rats treated with HAART. This is evidence of the neurotoxic effects of HAART that marked an increase in lipid peroxidase (MDA) resulting from excessive reactive oxygen species (ROS) production while inhibiting the production of antioxidant enzymes within hippocampal tissue. Consequently, the vulnerability of the hippocampal CA1 of the central nervous system to oxidative stress assault [61]. Studies [62, 63] have shown that HAART significantly increases oxidative stress levels beyond what HIV itself could cause.

This current study showed that HAART-loaded silver nanoparticles showed an improvement with antioxidant enzymes. The relatively small size of silver nanoparticles has a prospect in drug delivery by reducing the dosing frequency [25].

Hippocampal CA1 pyramidal cells are thought to be involved in memory and spatial learning [64]. The reduction in the neuronal cell synthetic role and evidence of weak Nissl staining characteristics of hippocampal CA1 neurons were observed in the diabetic group treated with HAART. In addition, the consequence of this neurotoxicity was seen in the behavioural studies and oxidative stress markers of the diabetic rat treated with HAART. This histological finding aligns with other studies [65, 66], who reported a strong association between pyramidal cell Nissl substance deficits and diabetes mellitus.

The HAART-AgNPs improved hippocampal pyramidal cell Nissl staining and a significant increase in the number of positive cells for Nissl staining compared to the diabetic group and diabetic rats treated with HAART. Silver nanoparticles are known for their antioxidant, antidiabetic, antiviral, and antifungal properties [67]. An improvement observed in these histological findings could be due to antioxidant and antidiabetic effects in the silver nanoparticles and the reducing the high-dose effect of HAART in the diabetic group treated with silver nanoparticle-HAART.

## 5. Conclusion

In this study, the administration of HAART to diabetic rats aggravates the complications of diabetes and promotes neurotoxic effects on the experimental rats, while HAART-loaded silver nanoparticle (nHAART) alleviates diabetes-induced neurotoxicity.

## Figures and Tables

**Table 1:** The effects of HAART and nHAART on GSH, SOD, and MDA in nondiabetic and diabetic rats.

Groups	Hippocampus oxidative stress markers		
	GSH (*μ*M/g)	SOD activity (*μ*M/mg)	MDA (*μ*M/mg)
Group A (ND control)	0.0814 ± 0.003	80.57 ± 2.338	0.2706 ± 0.035
Group B (ND+HAART)	0.0647 ± 0.004	72.56 ± 2.194	0.3508 ± 0.067
Group C (ND+HAART-AgNPs)	0.07457 ± 0.004	71.96 ± 3.973	0.3252 ± 0.062
Group D (diabetic control)	0.0274 ± 0.003^∗^	40.60 ± 4.284^∗^	0.6753 ± 0.023^∗^
Group E (D+HAART)	0.0347 ± 0.004	33.60 ± 5.178	0.6884 ± 0.031
Group F (D+HAART-AgNPs)	0.0629 ± 0.004^&$^	58.46 ± 4.985^$^	0.3820 ± 0.042^&$^

Data are expressed as mean ± SEM (*n* = 8); ^∗^*p* < 0.05 vs. group A (nondiabetic control), ^&^*p* < 0.05 vs. group D (diabetic control); ^$^*p* < 0.05 vs. group E (diabetic+HAART). ND = nondiabetic; D = diabetic.

## Data Availability

All data are included in the manuscript.
